# Research priorities of the European Society of Clinical Pharmacy (ESCP): a questionnaire-based study

**DOI:** 10.1007/s11096-025-01954-8

**Published:** 2025-06-20

**Authors:** Betul Okuyan, Martin C. Henman, Vibhu Paudyal, Anita E. Weidmann, Cathal Cadogan, Ankie Hazen, Daniela Fialová, Francesca Wirth, Monika Lutters, Bart Pouls, Zachariah Nazar, Fatma Al Raisi, Derek Stewart

**Affiliations:** 1 Clinical Pharmacy Department, Faculty of Pharmacy, Marmara University, Istanbul, Türkiye; 2 School of Pharmacy and Pharmaceutical Sciences, Trinity College Dublin, Dublin, Ireland; 3 Florence Nightingale Faculty of Nursing, Midwifery and Palliative Care, King’s College London, London, UK; 4 Department of Clinical Pharmacy, Innsbruck University, Innsbruck, Austria; 5 Department of General Practice and Nursing Science, Julius Center for Health Sciences and Primary Care, University Medical Center, Utrecht University, Utrecht, The Netherlands; 6 Department of Social and Clinical Pharmacy, Faculty of Pharmacy in Hradec Králové, Charles University, Hradec Králové, Czech Republic; 7 Department of Internal Medicine and Geriatrics, 1st Faculty of Medicine, Charles University, Prague, Czech Republic; 8 Department of Pharmacy, Faculty of Medicine and Surgery, University of Malta, Msida, Malta; 9 Cantonal Hospital Aarau, Aarau, Switzerland; 10 Department of Pharmacy, Pharmacology and Toxicology, Radboudumc, Nijmegen, The Netherlands; 11 Department of Clinical Pharmacy and Practice, College of Pharmacy, QU Health, Qatar University, Doha, Qatar; 12 Pharmacy Programme, Oman College of Health Sciences, Muscat, Oman; 13 Robert Gordon University, Aberdeen, UK; 14 Royal College of Surgeons, Dublin, Ireland

**Keywords:** Barriers, Clinical pharmacist, European Society of Clinical Pharmacy, Research priorities

## Abstract

**Introduction:**

It is important for health professional societies to involve members in defining their roles and future activities including research priorities.

**Aim:**

This study aimed to identify members’ views on the areas of research that European Society of Clinical Pharmacy (ESCP) should prioritise in delivering research support, research projects and education.

**Method:**

An online questionnaire was initially developed by the ESCP Research Committee and reviewed by the research team. It included structured and open-ended items related to respondents’ demographics, research experience, views on future research priorities, topics that ESCP should prioritise, and barriers to research involvement. After testing face and content validity, the questionnaire was sent to all ESCP members (N = 417). Descriptive statistics and summative content analysis were used.

**Results:**

Eighty-two responses were received (response rate: 19.7%). Research on real-world processes that facilitate the implementation of clinical pharmacy services into every-day practice was the priority for most respondents (n = 77, 93.9%). Respondents believed that ESCP should focus on research support for implementation science (n = 52, 63.4%) and methods to analyse clinical judgement and decision-making (n = 48, 58.5%). The perceived barriers to developing high-quality research in clinical pharmacy were reported as a lack of knowledge, skills and training, limited funding opportunities and insufficient time.

**Conclusion:**

Research topics identified will help to inform ESCP and its committees on the priorities for research activities of the society in the near future, as well as other collaborating professional organisations of the current priority research objectives of ESCP in the international context.

## Impact statements

- The study findings highlight a broad range of research areas that ESCP members believe the organisation should prioritise in terms of delivering research support, research projects and awarding future funding for clinical pharmacy research.
- ESCP and its committees can provide training and resources according to the research priorities and barriers to research identified in this study.
- Other professional organisations can take acount of these priorities for collaboration with ESCP or adapt them for future international collaborative pharmacy research.

## Introduction

Clinical pharmacy aims to optimise the use of medicines in order to achieve person-centred and public health goals through clinical pharmacy practice and research [1]. The scope of clinical pharmacy practice encompasses the cognitive, interpersonal and managerial activities undertaken by pharmacists across different settings relating to the appropriate selection, administration and monitoring of medicines by relevant stakeholders i.e. healthcare professionals, patients and the public [1]. Clinical pharmacy research, with its focus on optimising the use of medicines in preventing and managing disease and drug-therapy problems, contributes to generating the evidence that informs practicing clinical pharmacists, healthcare policy makers and other professionals and organisations responsible for the appropriate and safe use of medicines. Evidence generated from clinical pharmacy research has highlighted the vital contribution of pharmacists within health and social care systems and the variable scope of practice across different jurisdictions, such as vaccination provision during the COVID-19 pandemic [2–4], and the potential for pharmacists to undertake extended roles in areas such as prescribing [5, 6].

The European Society of Clinical Pharmacy (ESCP) is an international professional network of clinical pharmacists established in 1979. The vision of the ESCP is to advance quality and innovation in clinical pharmacy practice, education and research [7]. ESCP seeks to realise its vision relating to clinical pharmacy research by stimulating innovative and high-quality research in all areas of clinical pharmacy, promoting and enabling multi-centre research and disseminating findings from clinical research to support their uptake into clinical practice. These aims are also reflected in the Granada statements 2023 [8] and work of The International Collaboration of Pharmacy Journal Editors (ICPJE) [9] to identify the elements that may reinforce clinical and social pharmacy practice as a scientific discipline, i﻿mprove the quality of publications and advance the paradigms of related pharmacy practice research.

Since 2021, ESCP launched a call for Best Practice Paper submissions for publication in the International Journal of Clinical Pharmacy (IJCP) after a peer-review process [10]. These papers are related to development, evaluation and implementation of clinical pharmacy in practice and education, such as a pharmacist-led heart failure clinic, a quality improvement study for medication supply, and development of The Medicines Optimisation Innovation Centre (MOIC) in Northern Ireland.

ESCP has previously engaged with members regarding research capacity which refers to “the abilities of individuals, organisations and systems to undertake and disseminate high quality research effectively and efficiently” [13]. However, in order to achieve ESCP’s aim of promoting collaborative, innovative and high-quality research, it is vital to regularly identify the priority areas for future clinical pharmacy research, practice and education. Research priority exercises provide an important means of gathering insights from ESCP members and relevant stakeholders and establishing priority areas for future research [13]. Clinical pharmacy research surveys and further prioritisation of important research topics can also help to minimise research waste by ensuring that resources are strategically targeted towards priority areas [13, 14].

### Aim

This study aimed to identify ESCP members’ views on areas of research that ESCP should prioritise in terms of delivering education, research support and research projects, as well as awarding future research funding for clinical pharmacy research.

## Method

### Study design and study sample

The study is reported according to the checklist for reporting results of internet e-surveys (CHERRIES) [15]. In this questionnaire-based study, members of the ESCP at the time of survey (N = 417) were invited to participate between February and May 2024. No sample size was calculated according to the aim of the study. It was included all ESCP members. No financial incentive was provided to support this study.

### Questionnaire development

An online questionnaire was initially developed by the ESCP Research Committee and reviewed by the research team comprising members from all ESCP committees (including research, education, and communication committees) who are experienced pharmacists and researchers.

The inital list of potential research areas was generated and listed based on ESCP’s mission and vision statements [7] by two reseachers [AEW, MCH]. The respondents’ characteristics and research experience were collected. In order to determine the respondents’ self-reported receptiveness to change, a multiple-choice question that included four statements for each type of receptivity to change described by Rogers et al. was included [13, 16], namely whether responders were innovators, early adopters, early majority, late majority, or laggards. Respondents’ knowledge and attitudes to the ESCP Best Practice Papers published in IJCP were also collected.

A 5-point Likert scale was used to assess each area of research that ESCP should prioritise (1-strongly disagree to 5-strongly agree) and research related activities that ESCP should focus on in the future for the delivery of education and research support (1-not at all important to 5-very important). In addition, open-ended questions were included to enable respondents to suggest research areas, challenges in developing high-quality research in clinical pharmacy, priority topics in their research and suggestions for the priority topics that ESCP should prioritise in the future.

The research team reviewed the questionnaire for face and content validity, including evaluating each item to determine whether it measured what was intended and whether the list covered appropriate research areas in clinical pharmacy. No pilot study was conducted.

### Data collection

The online questionnaire was generated using Microsoft Forms® hosted by the University of Birmingham, UK. The first email, including a link to the consent form and the questionnaire, was sent out to 417 ESCP members by the International Office of ESCP in February 2024. There were no exclusion criteria.Two reminders were sent at four-week intervals via the social media accounts of ESCP. The survey was closed on May 24, 2024.

### Data analysis

Descriptive analysis (n, %), using Microsoft Excel 2025, was undertaken to report most frequent recommendations of research priority areas. Summative content analysis was used to analyse data from open-ended questions by two reseachers [BO, VP] [17].

### Ethics approval

The study was reviewed and approved by the ESCP General Committee and the University of Birmingham (UK) Research Ethics Committee (approval reference number RN_1098-Jun2023).

## Results

### Respondent characteristics

Eighty-two respondents completed the online questionnaire (response rate: 19.7%). Characteristics of the respondents are presented in Table 1. The majority of respondents were between 35 and 54 years (n = 62, 75.6%) and female (n = 60, 73.2%). Respondents were mostly from Europe (especially from the Netherlands [n = 8, 9.8%], United Kingdom [n = 8, 9.8%], Norway [n = 6, 7.3%], Switzerland [n = 6, 7.3%], Türkiye [n = 6, 7.3%] and 15 other European countries [n = 39, 47.6%]. Respondents from non-European countries [n = 7, 8.5%; Saudi Arabia, Argentina, Iraq, Jordan, Pakistan, Canada] also participated. The majority of respondents had a PhD degree (n = 58, 70.7%). More than half (n = 46, 56.1%) had practice experience of more than 15 years and the majority worked full-time (n = 66, 80.5%), especially in academia (n = 36, 43.9%). According to receptivity of change, the majority (n = 64, 78.0%) reported themselves as innovators and/or early adopters.

**Table 1:** Characteristics of respondents (n = 82)

	n (%)
Age (years)	
18–24	1 (1.2)
25–34	8 (9.8)
35–44	38 (46.3)
45–54	24 (29.3)
55–64	5 (6.1)
≥ 65	6 (7.3)
Gender	
Male	22 (26.8)
Female	60 (73.2)
Country of Practice	
The Netherlands	8 (9.8)
United Kingdom	8 (9.8)
Norway	6 (7.3)
Switzerland	6 (7.3)
Türkiye	6 (7.3)
Czech Republic	5 (6.1)
Sweden	5 (6.1)
Slovakia	5 (6.1)
Other countries*	31 (37.8)
Missing data	2 (2.4)
Postgraduate qualification	
Masters	33 (40.2)
Postgraduate certificate	17 (20.7)
Postgraduate diploma	12 (14.6)
PhD	58 (70.7)
Duration of practice as a pharmacist (excluding any career breaks)	
5 years or less	8 (9.8)
6–10 years	11 (13.4)
11–15 years	17 (20.7)
16–20 years	17 (20.7)
21–25 years	12 (14.6)
26–30 years	9 (11.0)
Over 30 years	8 (9.8)
Working schedule	
Full time	66 (80.5)
Part time	13 (15.8)
Other**	3 (3.7)
Main practice setting	
Academia	36 (43.9)
University hospital	21 (25.6)
Non-university hospital	10 (12.2)
Community pharmacy	8 (9.8)
Primary care medical practice	3 (3.7)
Industry, regulatory sciences, pharmaceutical policy	2 (2.4)
Other***	2 (2.4)
Receptivity to change	
Innovator (innovative with new ways of working)	41 (50.0)
Early adopter (serve as role model for others)	23 (28.0)
Early majority (think for some time before adopting new ways of working)	17 (20.7)
Laggards (resist new ways of working)	1 (1.2)
Late majority (cautious, tend to change once most peers have done so)	–

*Countries with less than five responses: Belgium, Italy, Ireland, Austria, Denmark, Portugal, Saudi Arabia, Spain, Argentina, Croatia, Germany, Iraq, Jordan, Malta, Pakistan, Poland, Romania, Canada; **not indicated; *** Both non university hospital and primary care medical practice (n = 1) and NHS Integrated Care Board (n = 1)

### Research experience

The research experience and practices of the respondents are presented in Table 2. From the respondents, 90.2% (n = 74) had been involved in research in the last two years and the majority (n = 52, 70.3%) were principal investigators and co-investigators. The majority (n = 67, 81.7%) had published research in a peer-reviewed scientific journal in the last two years. More than 70% of respondents had presented research at national (n = 49, 76.6% of 64 respondents) and international conferences (n = 50, 80.6% of 62 respondents) as a lead presenter in the last two years. Almost half of the respondents (n = 37, 45.1%) had supervised PhD students. The majority (n = 42, 51.2%) were members of a research committee and 36.6% (n = 30) stated they may consider submitting ESCP Best Practice Papers.

**Table 2:** Research practices of respondents (n = 82)

	n (%)
Involved in any research in the last 2 years (n = 82)	
Yes	74 (90.2)
No	8 (9.8)
Roles in research activities if they involved in any research in the last 2 years (n = 74)	
Principal investigator	52 (70.3)
Co-investigator	52 (70.3)
Other roles*	11 (14.9)
Published any research in a peer reviewed scientific journal in the last 2 years (n = 82)	
Yes	67 (81.7)
No	15 (18.3)
Presented research at a national conference (oral or poster communications) in the last 2 years (n = 82)	
Yes	64 (78.0)
No	18 (22.0)
Roles in presenting research in national conference (n = 64)	
Lead presenter	49 (76.6)
Co-presenter	35 (54.7)
Presented research at an international conference (oral or poster communications) in the last 2 year (n = 82)	
Yes	62 (75.6)
No	20 (24.4)
Roles in presenting research at an internation conference (n = 62)	
Lead presenter	50 (80.6)
Co-presenter	28 (45.2)
Missing data**	1 (1.6)
Supervised any research students in the last 2 year (n = 82)	
Undergraduate	35 (42.7)
Master level	55 (67.1)
PhD level	37 (45.1)
No	14 (17.1)
Missing data	2 (2.4)
Member of any research committee (such as in your workplace, University or professional/research/clinical society (n = 82)	
Yes	42 (51.2)
No	40 (48.8)
Knowledge and attitude for ESCP Best Practice Paper to International Journal of Clinical Pharmacy (n = 82)	
I may consider submitting ESCP Best Practice Papers in the future	30 (36.6)
I am aware about ESCP Best Practice Papers but have not considered submitting	24 (29.3)
I am not aware about ESCP Best Practice Papers and have not submitted it yet	16 (19.5)
Yes	9 (11.0)
Other***	3 (3.6)

*Including pharmacist (n = 2), assessors of grant application and involvement as a patient representative (n = 1), **stated as an individual studies (n = 1); ***not indicated (n = 1; 1.2%) and missing data (n = 2; 2.4%)

### Research priorities

Respondents’ opinions on areas of research that ESCP should prioritise in terms of delivery of education, research support, research projects and in awarding research funding for clinical pharmacy research in the future are presented in Table 3. The topics that had the highest level of agreement (strongly agree/agree) for prioritisation were: 1) research on real-world processes that facilitate the implementation of clinical pharmacy services into every-day practice (n = 77, 93.9%), 2) research evaluating the role of clinical pharmacists and their integration into the wider interdisciplinary team across all sectors (n = 74, 90.3%), and 3) research on assessing impact of emerging artificial intelligence (AI) on optimising patient care and clinical pharmacy processes (n = 72, 87.8%) and advancing the pharmacy workforce through education and training (n = 72, 87.8%).

**Table 3:** Respondents’ opinion on areas of research that ESCP should prioritise in terms of delivery of education, research support, delivering research projects and awarding any research funding for clinical pharmacy researchers in the future (n = 82)

	Strongly Disagree/Disagree n (%)	Neutral n (%)	Strongly agree/Agree n (%)
Clinical Pharmacy Services			
Identification of real-world processes that facilitate the implementation of clinical pharmacy services into every-day practice	1 (1.2)	4 (4.9)	77 (93.9)
Pharmacoeconomic studies (balancing the cost and benefits of interventions with limited resources)	3 (3.6)	18 (22.0)	61 (74.4)
Best practice developments in non-medical prescribing/deprescribing services	-	13 (15.8)	69 (84.2)
Advances in medicine information services	2 (2.4)	24 (29.3)	56 (68.3)
Exploring the impact of pharmacy-based public health service provision across Europe and the role of the pharmacist as public health provider	2 (2.4)	11 (13.4)	69 (84.2)
Addressing health inequalities and supply of medicines	3 (3.7)	26 (31.7)	53 (64.6)
Ways to develop environmentally sustainable pharmacy services	4 (4.9)	22 (26.8)	56 (68.3)
Effect of human factors/ergonomics on health system resilience	10 (12.2)	32 (39.0)	40 (48.8)
Clinical Pharmacy Team			
Advancing the role of the pharmacy technician within the wider pharmacy and interdisciplinary team	4 (4.9)	30 (36.6)	48 (58.5)
Impact of hierarchical and traditional forms of working on the reform of healthcare practice	3 (3.7)	38 (46.3)	41 (50.0)
Evaluating the role of clinical pharmacists and their integration into the wider interdisciplinary team across all sectors	1 (1.2)	7 (8.5)	74 (90.3)
Advancing the pharmacy workforce through education and training	-	10 (12.2)	72 (87.8)
Medication safety			
Effect of medication reconciliation on patient safety; downstream processes; economic costing and operational impact	3 (3.6)	17 (20.7)	62 (75.6)
Effect of medication reviews/analysis on patient safety; downstream processes; economic costing and operational impact	2 (2.4)	9 (11.0)	71 (86.6)
Impact of pharmacogenomics, pharmacogenetics and personalized medicine on patient safety and feasibility of its implementation into practice	1 (1.2)	13 (15.9)	68 (82.9)
Influence of health literacy on patient safety; downstream processes; economic costing and operational impact	2 (2.4)	20 (24.4)	60 (73.2)
Assessment of pharmacovigilance reports to improve current medication safety practice	8 (9.8)	27 (32.9)	47 (57.3)
Patient safety at transitions of care points & high-risk situations	–	12 (14.6)	70 (85.4)
Advances in the contribution of the pharmacist in infection prevention, control and antimicrobial stewardship	–	14 (17.1)	68 (82.9)
Efficacy, effectiveness, efficiency and safety of medicines, pharmacotherapy and related medical devices	3 (3.6)	11 (13.4)	68 (82.9)
Advances in and impact of adherence strategies and supporting technologies	3 (3.6)	14 (17.1)	65 (79.3)
Digital Health			
Health technology assessment studies to improve the uptake of cost-effective innovative technologies	4 (4.9)	15 (18.3)	63 (76.8)
Use of modern technological solutions to improve administration, adherence, and patient education	1 (1.2)	15 (18.3)	66 (80.5)
Impact of emerging artificial intelligence systems on optimising patient care and clinical pharmacy processes	1 (1.2)	9 (11.0)	72 (87.8)
Analysis of large and complex data sets of service data, clinical data and clinical/disease registries – Big Data	3 (3.6)	13 (15.9)	66 (80.5)
Facilitators and consequences of virtual reality worlds to purchase medication and provide virtual consultations with patients—Metaverse Pharmacies	8 (9.8)	32 (39.0)	42 (51.2)
Policy and Education			
Clinical pharmacy and medicines related policy and guideline development	4 (4.9)	12 (14.6)	66 (80.5)
Clinical pharmacy related education, training and professional development	1 (1.2)	11 (13.4)	70 (85.4)

According to summative content analysis of open-ended items, topics with the reported highest research priority were clinical pharmacy services, geriatrics, transition of care, mental health, medication adherence, medication safety, drug policy, implementation science, patient education, drug safety and involvement, prescribing, personalised medicine, pharmacogenomics (PGx) and therapeutic drug monitoring (TDM), education and training, digital health and big data (Table 4). Suggested novel research areas included: astropharmacy, mental health, guideline development, interdisciplinary cooperation between clinical pharmacists and other health care providers, communication with patients and involvement of patients in research (Table 4).

**Table 4:** Themes and illustrative quotes related to their self-reported priority topics in their research and suggestions for the priority topics that ESCP should prioritise in the future

	Examples for the respondents’ priority topics for their own clinical pharmacy research	Examples for the respondents’ suggestions about research areas that ESCP should prioritise in the future	Examples for the respondents’ suggestions about other research training areas that ESCP should prioritise
Clinical Pharmacy service	“Pharmacotherapy optimization” “Advanced scope of practice” “Medication review” “Antibiotic stewardship” “Oncology pharmacy” “Supportive care” “Chronic pain and opioid use”	“Differences in worldwide pharmacists’ scope of practice and its future” “Health economics related to a clinical pharmacists’ interventions” “Defining clinical pharmacy key performance indicators” “Astropharmacy (in which we can explore the role of pharmacist in space) and also the establishment of Space Pharmacy Council” “Work-related burnout and stress in clinical pharmacists;” “Medication outcome research in high-risk patients (outside the Evidence Based Medicine medicine quidelines)” “Clinical pharmacy for disadvantaged and vulnerable populations.”	
Geriatrics	“Population ageing, importance of specific geriatric clinical pharmacy services in different settings of care” “Safety and efficacy of geriatric pharmacotherapy” “Polypharmacy” “Deprescribing”	“Geriatric clinical pharmacy”	
Transition of care	“Discharge process (medication information transfer)”	“The role of pharmacists in patients’ care continuity”	
Mental health	“Unnecessary or unnecessarily long use of psychopharmaceuticals. Reducing and stopping medication. Patient counselling in decision-making about choices in care. Patient counselling on reducing and stopping psycho-pharmaceuticals.” “Broad area of psychotropic drug use in mental health hospital”	“Mental health”	
Medication adherence	“Adherence anticoagulation in heart failure”		
Medication safety	“Good medication administration practice (enteral feeding tubes, parenteral administration,…)”	“Contamination of the surfaces, Closed System Transfer Device”]	
Policy	“Policy development and advocacy” “Bioethics” “Public Health” “Health Inequalities” “Policy and Education” “Clinical rules”	“Guidelines for interdisciplinary cooperation between clinical pharmacists, doctors, nurses and other stakeholders (including the government, healthcare providers, insurance representatives …)” “Global health” “Philosophy of health science with focus on the role of pharmaceuticals and pharmacists” “Population Health specific data driven outcomes to support the delivery of medicines optimisation in clinical services.”	“Law in clinical pharmacy research”
Implementation science	“Implementation research” “Implementation of clinical pharmacist in interdisciplinary teams” “The impact of a clinical pharmacist on health care system, for patients, for health care professionals. What are the benefits of implementing clinical pharmacists in health care wards? There is a lack of qualitative research.” “Clinical Pharmacy Services (?) implementation research and pharmacoeconomic evaluations”]		“Implementation science in settings of care where development of clinical pharmacy is needed “ “Sustainable pharmacy and medicines use processes”
Patient education, safety and involvement	“Patient and public involvement in healthcare research “ “Patient empowerment” “Pharmacy service and communication with patients”	“How to behave to patients as a clinical pharmacist, particularly to children, geriatric or psychiatric patients. We should know how to give specific information about the drug and the treatment.” “Communication with patients—how to do shared decision making—Education of patient and patient communication” “Co-production with patients”	
Prescribing	“Non-Medical prescribing” “Pharmacist prescribing”	“Prescribing”	
Personalised medicine, pharmacogenomics (PGx) and Therapeutic Drug Monitoring (TDM)	“Pharmacogenetics, biomarkers of medication effectiveness and safety/toxicity, cardiovascular pharmacotherapy, …” “Implementation of PGx “TDM in Psychiatry” “Antibiotic TDM”	“Pharmacogenomics”	“Clinical Pharmacology”
Education and Training	“Assessment of CP performance” [CP – Clinical Pharmacy] “Training and deploying pharmacy technicians in CP” “Pharmacy education” “Prioritise trainings on a clinical pharmacy specialist topic (general trainings e.g. systematic reviews are broadly available)” “Interprofessional workplace-based learning” “Clinical decision-making education and interprofessional education (IPE)”		“Interprofessional learning”
Digital Health and Big Data	“Development and Impact of clinical decision support systems (CDSS)” “Using AI (artificial intelligence) applications in research” “Application of e-health in proper medication use” “Digitalisation of medication management in hospitals and community care” “Adherence enhancing technology” “Non-randomised routinely collected health data (big data) processing”	“Clever, more complex clinical decision support systems with pharmacist involvement” “Learning methods, clinical impact of CDSS” “Digital tools for check of medication appropriateness” “Telehealth and mobile health”	“Training in application of AI in clinical pharmacy research (strenghts, limitations)” “References endnote “
Research methods			“Data management” “Innovative research methods” “Critical assessment of EBM research and practice.”

### Expectations from ESCP

Respondents’ opinions on research-related activities that ESCP should focus on in the future for the delivery of education and research support are presented in Table 5. The majority thought that ESCP should focus on the delivery of education and research support for implementation science (n = 52, 63.4%) and methods to analyse clinical judgement and decision-making (n = 48, 58.5%). According to summative content analysis of open-ended items, suggested new topics were interprofessional learning, as well as education and training in light of AI in clinical pharmacy research (Table 4).

**Table 5:** Respondents’ opinion on research related activities that ESCP should be focusing on in the future for the delivery of education and research support (n = 82)

	Not at all important n (%)	Somewhat important n (%)	Neutral n (%)	Important n (%)	Very important n (%)
Patient and public involvement in all health-related research teams	3 (3.7)	21 (25.6)	16 (19.5)	22 (26.8)	20 (24.4)
Cross-geographical and multisectoral initiatives for patient safety	5 (6.1)	13 (15.9)	24 (29.3)	24 (29.3)	16 (19.5)
Training in quantitative research methods	7 (8.5)	15 (18.3)	17 (20.7)	27 (32.9)	16 (19.5)
Training in qualitative research methods	11 (13.4)	12 (14.6)	14 (17.1)	26 (31.7)	19 (23.2)
Training in systematic reviews and meta-analyses	7 (8.5)	16 (19.5)	20 (24.4)	20 (24.4)	19 (23.2)
Methods to analyse clinical judgement and decision-making	6 (7.3)	16 (19.5)	12 (14.6)	21 (25.6)	27 (32.9)
Implementation research	8 (9.8)	14 (17.1)	8 (9.8)	23 (28.0)	29 (35.4)
Training in academic writing	7 (8.5)	14 (17.1)	23 (28.0)	23 (28.0)	15 (18.3)
Research project management	8 (9.8)	13 (15.9)	20 (24.4)	22 (26.8)	19 (23.2)

### Barriers in research studies

Respondents’ self-reported barriers regarding development of high-quality research in clinical pharmacy are presented in Table 6. Respondents reported many barriers in developing high-quality research in clinical pharmacy including lack of knowledge, skills and training, limited funding opportunities and resources, insufficient time, insufficient collaboration and social support, barriers related to beliefs on consequences and ethical issues. Respondents also reported barriers related to regulations, conducting international studies and designing research studies.

**Table 6:** Themes and illustrative quotes related to their self-reported barriers in developing high-quality research in clinical pharmacy

Themes	Example of responses
Lack of knowledge, skills and training	“Workforce expertise” “Lack of capacity in pharmacy researchers to go for interdisciplinary grants Maybe that there are only few pharmacists who have really good research skills/experience to provide very high-quality research?”
Limited funding opportunities and resources	“Funding and priorities” “Lack of resources” “Resources (manpower, finances)” “Lack of professional journals with a higher impact…”
Insufficient time	“Time required by hospital pharmacy teams, which are often already suffering from a shortage of skilled labour.” “Time investment from pharmacist” “It's difficult to get hard data such as cost savings and reduced Emergency Department visits. Time consuming and complex research. But important!”
Barriers related to regulations	“Lack of support locally governance” “Reimbursement” “Regulatory hurdles” “Time and legal challenges that makes it harder for us to make students help us in research (they cannot use the patients’ medical charts for example in evaluating research)”
Barriers related to conducting international studies	“Diversity of situation of clinical pharmacy services across Europe” “Standardisation of practices, differences in training and healthcare systems.” “Absence of integrated advanced practices” “Interdisciplinary cost-effectiveness research at an international level” “International research in implementation science, particularly in "difficult" settings of care (e.g. nursing homes, home care, General Practitioner ambulances)…”
Insufficient collaboration and social support	“Interprofessional/interdisciplinary collaboration and transition of care situations” “Clinicians are often too busy with clinical practice and do not have time or willingness to participate in data acquisition.” “Lack of social support”
Barriers in designing research studies	“Reasonable sample size –> multi-center studies + multi-country studies “ “Have a well-designed methodology” “Hard to target the population that can benefit most from the research (for example health literacy)” “Choosing the right outcome measures, there are none that fit perfectly in my opinion.” “Obtaining accurate and relevant data from clinical settings” “Accessibility of quality clinical data”
Barriers related to beliefs on consequences	“Translation of the results as well as their uptake in multidisciplinary guidelines (not only in those of pharmacists)” “The traditional weighting of scientific evidence according to the EBM model is under pressure. A transition is underway towards patient-centred individualised pharmaceutical care delivery with shared decision making and seamless care as the basis. The challenge for ESCP in this transition is to critically evaluate the usual scientific methodologies for their appropriateness in the changing relationships between patients and medical/pharmaceutical professionals. Such an evaluation can be undertaken from a philosophy-of-science perspective. There are already good examples of this in psychiatry. For diagnostics, we see a shift towards phenomenological psychopathology there. This involves connecting to established scientific practices.”
Ethical issues	“General Data Protection Regulation (GDPR) aspects”

## Discussion

Respondents recognised the importance of a broad range of topics across areas including clinical pharmacy services, medication safety and digital health. The areas that achieved the highest level of agreement for prioritisation focused on identifying real-world processes that facilitate the implementation of clinical pharmacy services into every-day practice, evaluating the role of clinical pharmacists and their integration into the wider interdisciplinary team across all sectors, assessing impact of emerging AI on optimising patient care and clinical pharmacy processes and advancing the pharmacy workforce through education and training. Respondents believed that ESCP should focus on research support for implementation science, methods to analyse clinical judgement and decision-making and training in quantitative research methods. Key perceived barriers to developing high-quality research in clinical pharmacy included lack of knowledge, skills and training, limited funding opportunities and resources, and insufficient time as indicated in a survey concerning pharmacists and clinical research [18].

Advancing quality and innovation in clinical pharmacy education, practice and research is a key vision of ESCP [7]. As part of their strategic vision, other professional pharmacy organisations have also prioritised the need to promote research. For example, the International Pharmaceutical Federation (FIP) emphasises the need for pharmacists to take on significant responsibilities in research to contribute to improvements in global health by advancing drug discovery and development, clinical practice and education [19]. The findings from this study provide valuable information on research areas of importance for clinical pharmacy.

The areas identified in this study that achieved the highest level of agreement for prioritisation highlight some long-standing issues that have not been adequately addressed by previous research to-date. For example, issues around the implementation of clinical pharmacy services into every-day practice were previously highlighted in another ESCP survey which emphasised widespread variability in clinical pharmacy practice patterns across 40 European countries [20]. This variability also creates challenges in addressing other priority areas identified in this study, such as evaluating the role of clinical pharmacists and their integration into the wider interdisciplinary team across all sectors. Contributing factors to this variability may be the current scope of practice of pharmacists [21] and the capacity for health research in low- and middle-income countries highlighted in other studies [22]. The FIP Pharmacy Practice Research Special Interest Group proposed more specific topics focused on evaluating the impact of new therapies and vaccines and/or pharmacy services and education during the COVID-19 pandemic [23]. The American College of Clinical Pharmacy (ACCP) strategic plan for 2024–2026 promotes research studies and early caregiver researchers, without specifying priority research areas [24]. With respect to Middle Eastern Arab countries, Obaid et al. [25] suggested setting research priorities on a country-by-country basis.

In terms of addressing issues around clinical pharmacy-related education, training and professional development, ESCP takes active measures in addressing these areas through regular webinars that are open to members and non-members [26]. The webinars have, to-date, covered a range of clinical topics such as antimicrobial stewardship, PGx and TDM, and research topics such as survey research, writing a manuscript for publication in a peer-reviewed scientific journal, and research using AI methods. The ESCP annual symposium and spring workshops also provide important opportunities for continuing professional development for clinical pharmacists and researchers. For example, the pre-conference Masterclass at the 2024 ESCP annual symposium focused on implementation science in clinical pharmacy (*Getting started with Implementation Science in Clinical Pharmacy*). The study findings provide useful information for future training programmes in areas such as AI, clinical judgement and decision-making.

The questionnaire’s free text responses highlighted important new topics and emerging areas of interest, such as training in the application of AI in clinical pharmacy research. Since the launch of ChatGPT in late 2022, the role of AI in clinical pharmacy education and research continues to be debated. As the technology continues to evolve, continuous research will be needed to investigate the positive and negative implications of AI on clinical pharmacy practice, research and education [27]. The ESCP Research Committee actively participates in the organisation of the European training school for young postgraduate researchers on this topic, titled “*Artificial intelligence (AI) in the Healthcare, Clinical Pharmacy and Optimization of Pharmacotherapy: New Advances in Research and Clinical Practice” (*April–May, 2025).

Another novel area that was suggested was astropharmacy, which is an area of pharmacy that addresses effective medical and pharmaceutical care in the context of space exploration [28]. This area will likely be of increasing interest in the years and decades ahead, and poses a range of unanswered research questions of relevance to clinical pharmacy, such as the implications of changes in pharmacokinetics and pharmacodynamics on the safe use of medicines during space exploration, as well as issues relating to drug stability [29].

The main strength of this study is that it identified views on priorities for clinical pharmacy research from an internationally diverse sample of ESCP members across 26 countries, and mostly PhD researchers intensively involved in clinical pharmacy research. The main limitation of the study was the response rate (19.7%) which was slightly higher than in a previous ESCP members survey (16.7%) [13]. Although, there is no consensus about the minimum acceptable rate of online survey, Wu et al. [30] reported the response rate as 44% for education related studies. The largest proportion of respondents were based in academic settings (43.9%), meaning that the identified priorities may not be fully reflective of those working in clinical practice settings. This limitation of the study should be considered in the context of conducting practice-based research. It must also be noted that the inclusion of pre-specified priorities may have influenced the respondents’ selection process. However, free-text response options were included to enable respondents to include any areas of importance that were not listed. The lack of pilot testing of the questionnaire was another limitation of the study.

Although the study findings have highlighted a broad range of areas and topics that ESCP members perceived to be important for future research, further research is needed to identify specific research questions that need to be addressed within each area. This could include consultation exercises with key stakeholders including clinical pharmacists and clinical pharmacy researchers, as well as those representing other health professions with active links to clinical pharmacy. Any future research activities, including funding applications, should also consider having Patient and Public Involvement (PPI) representation to improve the quality, efficiency, and impact of clinical pharmacy research, and ensure that those affected by the research are given the opportunity to provide input towards what research is conducted and how it is undertaken [31].

The ESCP Research Committee members work to develop and implement guidelines and educational materials to support ESCP members in their research, including annual webinars, several “How to” papers on ESCP website providing guidance for ESCP members such as on how to appropriately write a scientific abstract and prepare scientific presentations, as well as delivering Masterclasses, various workshops and other training events on clinical pharmacy research (e.g. training schools). The Research Committee published two commentaries to improve the quality of writing in a research paper and successful grant applicatio, as well as a systematic review of available clinical pharmacy practice guidelines. ESCP and its Research Committee will continue to provide training and resources according to the research priorities and barriers to research identified in this study.

## Conclusion

The study findings highlight a broad range of areas of research that ESCP members believe the organisation should prioritise in terms of delivering education, research support, research projects and awarding future funding for clinical pharmacy research. Areas of particular importance to respondents to this survey included real-world processes that facilitate the implementation of clinical pharmacy services into routine practice, evaluating the role of clinical pharmacists and their integration into the wider interdisciplinary team across all sectors, assessing the impact of artificial intelligence on optimising patient care and clinical pharmacy processes, and advancing the pharmacy workforce through education and training. Training and resources are needed to support ESCP members and the wider network of clinical pharmacists and clinical pharmacy researchers to engage in high quality research to advance the evidence base in these areas, as well as new and emerging areas of interest such as the application of artificial intelligence and astropharmacy topics in clinical pharmacy research.

## Data Availability

The data are available from the authors on request.
