# Correction: Research priorities of the European Society of Clinical Pharmacy (ESCP): a questionnaire-based study

**DOI:** 10.1007/s11096-025-02067-y

**Published:** 2025-12-05

**Authors:** Betul Okuyan, Martin C. Henman, Vibhu Paudyal, Anita E. Weidmann, Cathal Cadogan, Ankie Hazen, Daniela Fialová, Francesca Wirth, Monika Lutters, Bart Pouls, Zachariah Nazar, Fatma Al Raisi, Derek Stewart

**Affiliations:** 1 Clinical Pharmacy Department, Faculty of Pharmacy, Marmara University, Istanbul, Turkey; 2 School of Pharmacy and Pharmaceutical Sciences, Trinity College Dublin, Dublin, Ireland; 3 Florence Nightingale Faculty of Nursing, Midwifery and Palliative Care, King’s College London, London, UK; 4 Department of Clinical Pharmacy, Innsbruck University, Innsbruck, Austria; 5 Department of General Practice and Nursing Science, Julius Center for Health Sciences and Primary Care, University Medical Center, Utrecht University, Utrecht, The Netherlands; 6 Department of Social and Clinical Pharmacy, Faculty of Pharmacy in Hradec Králové, Charles University, Hradec Králové, Czech Republic; 7 Department of Internal Medicine and Geriatrics, 1st Faculty of Medicine, Charles University, Prague, Czech Republic; 8 Department of Pharmacy, Faculty of Medicine and Surgery, University of Malta, Msida, Malta; 9 Cantonal Hospital Aarau, Aarau, Switzerland; 10 Department of Pharmacy, Pharmacology and Toxicology, Radboudumc, Nijmegen, The Netherlands; 11 Department of Clinical Pharmacy and Practice, College of Pharmacy, QU Health, Qatar University, Doha, Qatar; 12 Pharmacy Programme, Oman College of Health Sciences, Muscat, Oman; 13 Robert Gordon University, Aberdeen, UK; 14 Royal College of Surgeons, Dublin, Ireland

**Correction to: International Journal of Clinical Pharmacy (2025) 47:1770–1783** 10.1007/s11096-025-01954-8

In this article, the following references and their corresponding in-text citations were removed during the editorial review post publication.

11. Thomas A, Forsyth P, Griffiths C, et al. Implementation and evaluation of pharmacist-led heart failure diagnostic and guideline directed medication therapies clinic. Int J Clin Pharm. 2024;46(6):1247–55.
12. Tait LA, Cassidy N, Jamieson D, et al. Medication supply at hospital discharge via community pharmacy: a quality improvement study. Int J Clin Pharm. 2023;45(6):1309–16.
13. Hogg A, Scott M, Fleming G, et al. The Medicines Optimisation Innovation Centre: a dedicated centre driving innovation in medicines optimisation-impact and sustainability. Int J Clin Pharm. 2024;46(5):1001–9.
35. Weidmann AE, Cadogan CA, Fialová D, et al. How to write a successful grant application: guidance provided by the European Society of Clinical Pharmacy. Int J Clin Pharm. 2023;45(3):781–6.
36. Wirth F, Cadogan CA, Fialová D, et al. Writing a manuscript for publication in a peer-reviewed scientific journal: guidance from the European Society of Clinical Pharmacy. Int J Clin Pharm. 2024;46(2):548–54.
37. Paudyal V, Okuyan B, Henman MC, et al. Scope, content and quality of clinical pharmacy practice guidelines: a systematic review. Int J Clin Pharm. 2024;46(1):56–69

The original article has been corrected.

